# Vitamin D, periodontitis and tooth loss in older Irish adults

**DOI:** 10.1017/S000711452400148X

**Published:** 2024-08-28

**Authors:** Lewis Winning, Siobhan Scarlett, Michael Crowe, Michael O’Sullivan, Rose Anne Kenny, Brian O’Connell

**Affiliations:** 1 Dublin Dental University Hospital, Trinity College Dublin, Dublin D02 F859, Republic of Ireland; 2 The Irish Longitudinal Study on Ageing (TILDA), Trinity College Dublin, Dublin, Republic of Ireland; 3 Mercer’s Institute for Successful Ageing, St James’s Hospital, Dublin, Republic of Ireland

**Keywords:** Vitamin D, 25-hydroxyvitamin D, Periodontitis, Tooth loss, Ageing

## Abstract

The aim of this study is to investigate whether 25-hydroxyvitamin D (25(OH)D) is associated with periodontitis and tooth loss in older adults. A total of 2346 adults underwent a detailed dental examination as part of the health assessment of a national population study – The Irish Longitudinal Study of Ageing. 25(OH)D analysis was performed on frozen non-fasting total plasma using LC-MS. The analysis included both multiple logistic regression and multinominal logistic regression to investigate associations between 25(OH)D concentration, periodontitis and tooth loss, adjusting for a range of potential confounders. Results of the analysis found the mean age of participants was 65·3 years (sd 8·2) and 55·3 % of the group were female. Based on the quintile of 25(OH)D concentration, participants in the lowest *v*. highest quintile had an OR of 1·57 (95 % CI 1·16, 2·13; *P* < 0·01) of having periodontitis in the fully adjusted model. For tooth loss, participants in the lowest *v*. highest quintile of 25(OH)D had a RRR of 1·55 (95 % CI 1·12, 2·13; *P* < 0·01) to have 1–19 teeth and a RRR of 1·96 (95 % CI 1·20, 3·21; *P* < 0·01) to be edentulous, relative to those with ≥ 20 teeth in the fully adjusted models. These findings demonstrate that in this cross-sectional study of older men and women from Ireland, 25(OH)D concentration was associated with both periodontitis and tooth loss, independent of other risk factors.

Periodontitis is a multifactorial inflammatory disease associated with dysbiotic dental plaque biofilms and characterised by progressive destruction of the tooth-supporting structures^(1)^. The global age-standardised prevalence of severe periodontitis was 9·8 % (95 % uncertainty interval 8·2 %–11·4 %) in 2017, whilst the number of prevalent cases was 796 million (95 % uncertainty interval 671–930 million) worldwide^(2)^. Untreated periodontitis is a major cause of tooth loss in adults^(3)^, which may lead to masticatory dysfunction that may affect nutrition, quality of life and self-esteem, as well as having socio-economic impacts^(4,5)^. Total tooth loss (edentulism) is a particularly debilitating and challenging condition. Although global edentulism levels have decreased in recent decades, prevalence remains high amongst older individuals^(6)^.

Vitamin D plays a crucial role in regulating bone metabolism, which is directly relevant to the support and maintenance of dental structures^(7)^. It enhances the absorption of Ca and phosphate, minerals essential for healthy bone and tooth structure^(8)^. Furthermore, vitamin D possesses anti-inflammatory properties, which are significant in mitigating the inflammatory response in periodontitis. It downregulates the production of pro-inflammatory cytokines and upregulates anti-inflammatory cytokines, thereby potentially reducing the severity of periodontal inflammation^(9)^. Vitamin D also modulates the immune response that enhances the body’s ability to combat oral pathogens that contribute to periodontitis^(10,11)^. Therefore, inadequate vitamin D levels may potentially compromise periodontal health and contribute to the progression of periodontitis and subsequent tooth loss^(12)^.

The primary sources of vitamin D are dietary intake and sunlight exposure in the form of vitamin D_2_ and D_3_, which are metabolised to 25-hydroxyvitamin D (25(OH)D) in the liver^(13)^. In Ireland, vitamin D synthesis via sun exposure is particularly limited due to the country’s northern latitude. It has been estimated that approximately half of Ireland’s general population has a 25(OH)D level < 50 nmol/l^(14)^. Vitamin D levels have also been investigated in older adults in Ireland. In a large nationally representative study, the prevalence of vitamin D deficiency, defined as 25(OH)D < 30 nmol/l, was found to be 13·1 % (95 % CI 12·1, 14·2) amongst 5356 community-dwelling older adults^(15)^. Negative correlates of 25(OH)D concentration included smoking, geographic location, time of year of testing (low vitamin D synthesis period), obesity, physically inactive and older age. A further study investigating three distinct disease cohort groups from Ireland (*n* 1895, 1233 and 1316) reported vitamin D deficiencies (25(OH)D < 30 nmol/l) ranging from 13·8 to 43·6 % in older unsupplemented adults^(16)^. This highlights the importance of co-morbidities and vitamin D status in older adults.

Previous epidemiological studies have suggested low levels of vitamin D as a risk factor for periodontitis^(17)^; however, a causal relationship remains uncertain^(18)^. Existing observational studies have limitations including small sample size and insufficient adjustment of important covariates^(19)^. A recent systematic review and meta-analysis were conducted to explore vitamin D levels amongst periodontitis cases in comparison with periodontally healthy cases^(20)^. Eleven studies were selected to perform meta-analysis on, with the majority of these studies being the case–control design type studies with < 100 participants. Periodontitis cases were found to have a decreased circulating 25(OH)D level compared with the healthy controls (pooled mean difference = –6·80, 95 % CI –10·59, –3·02). Heterogeneity was high (*I* ^2^ = 97 %), and nearly all studies included were found to have a high risk of bias.

The aim of this study, therefore, was to investigate the association between 25(OH)D concentration and periodontitis/tooth loss in a large group of older adults from Ireland.

## Methodology

### Population

Subjects were recruited from the Irish Longitudinal Study of Ageing (TILDA), which is a nationally representative, large prospective cohort study on the social, economic and health circumstances of community-dwelling adults aged 50 years and older in Ireland. Details of TILDA’s design including survey methodology and weighting scheme have previously been described^(21,22)^. Briefly, the study comprises a clustered stratified random sample of the community-dwelling population aged ≥ 50 years old. There are three components to data collection: a computer-assisted personal interview administered by trained social interviewers in the participants’ own homes, a self-completion questionnaire completed in the participants’ own time and a comprehensive health assessment in a dedicated health centre including blood sampling. Data on a broad range of domains including health status, healthcare utilisation and demographic, social and economic circumstances are all collected at successive ‘waves’ performed biennially.

**Fig. 1.**
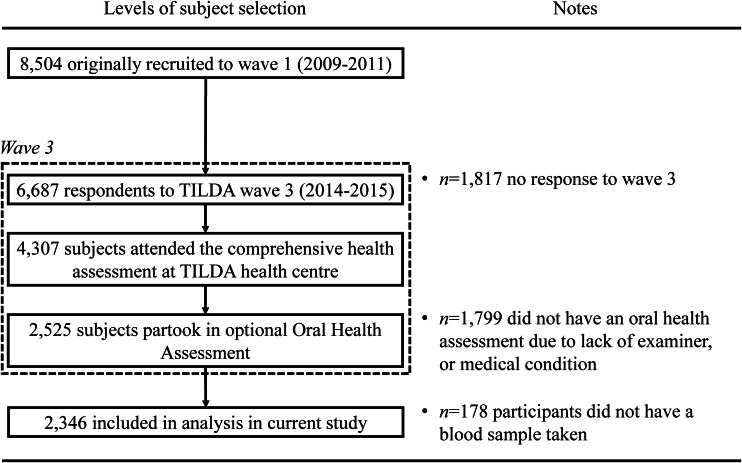
Recruitment and enrolment of study participants.

Fieldwork at Wave 1 (2009–2011) yielded a sample of 8504 adults. At Wave 3 (2014–15), the response yielded a sample of 6902. Of this sample, 4307 attended the comprehensive health assessment at the TILDA health centre, Trinity College Dublin. Alongside the comprehensive health assessments, subjects were offered an optional oral health assessment in which 2525 participants took part. This study is based on a cross-sectional analysis of the 2346 subjects who had blood sampling carried out and attended the oral health assessment at Wave 3 (Fig. 1).

This study was conducted according to the guidelines laid down in the Declaration of Helsinki, and all procedures involving human subjects/patients were approved by the Research Ethics Committee of the Faculty of Health Sciences, Trinity College Dublin (Wave 3, reference: ‘Main Wave 3 Tilda Study’, date of approval: June 2014). Written informed consent was obtained from all subjects/patients.

### Oral health assessment

The oral health assessment was based on the WHO epidemiological survey methodology^(23)^. All oral health assessments were completed by one of four trained dentists who had been calibrated against a ‘gold standard’ set by a senior clinical researcher prior to the study commencing. This involved a calibration exercise performed by all examiners on eight non-participant volunteers at the outset of the study^(24)^. Repeat examinations were performed until discrepancies between the gold standard examiner and other assessors were resolved and met an accepted clinical standard (κ agreement values of > 0·8). Regular meetings took place throughout the duration of the study to ensure inter- and intra-examiner consistency and reproducibility.

Periodontal status was evaluated utilising the Community Periodontal Index (CPI) partial mouth recording protocol^(25)^. Index teeth examined were the maxillary and mandibular left and right first and second molars, the maxillary right central incisor and the mandibular left central incisor (10 teeth). If no index teeth or tooth was present in a sextant qualifying for examination, all the remaining teeth in that sextant were examined, and the highest score was recorded as the score for that sextant. If only one tooth remained in a sextant, it was included in the examination of the adjacent sextant. Periodontal probing was carried out utilising a WHO CPITN (E) probe (Hu-Friedy), by inserting the probe tip into the periodontal pocket and exploring the extent of pocketing on all surfaces of the tooth (distobuccal to mesiobuccal and distopalatal to mesiopalatal) keeping the probe parallel to the long axis of the tooth. The scoring criteria were Code 0, no findings of gingival inflammation; Code 1, bleeding evident after probing; Code 2, calculus deposits (including those detected by probing up to 4 mm beneath the gingival margin); Code 3, depth of periodontal pocket ≥ 4 mm and < 6 mm; Code 4, depth of periodontal pocket ≥ 6 mm; and Code X, sextant excluded (less than two teeth present). The absence or presence of periodontitis was dichotomised as CPI 0–2, and CPI 3 and 4, respectively.

Tooth presence was recorded for each of the thirty-two teeth to calculate the number of natural teeth present in each adult. The examiners used clinical judgement regarding tooth morphology and took into account the respondent’s previous dental history if a doubt existed as to the correct notation for a particular missing tooth. As well as the total number of natural teeth present, participants were also categorised into three groups to reflect tooth loss status: ≥ 20 teeth, 1–19 teeth and edentulous.

### 25-hydroxyvitamin D concentration

Frozen, non-fasting total plasma samples were accessed for the blood biomarker measurements. The concentration of total 25-hydroxyvitamin D (25(OH)D) (including D_2_ and D_3_) was quantified by LC-MS with a validated method (Chromsystems Instruments and Chemicals GmbH; MassChrom 25-hydroxyvitamin D_3_/D_2_) in the Biochemistry Department of St James’s Hospital (accredited to ISO 15189 standard). Before 25(OH)D was analysed on the mass spectrometer, there was extensive clean-up of the samples with steps including protein precipitation, filtration and further chromatography on the mass spectrometer itself, reducing the risk of potential issues, for example, micro-clots. The quality and accuracy of the method were monitored using internal quality controls, participation in the Vitamin D External Quality Assessment Scheme and the use of the National Institute of Standards and Technology 972 vitamin D standard reference material. The respective inter- and intra-assay coefficients of variation were 5·7 and 4·5 %. Low and high vitamin D synthesis periods were defined as winter-spring and summer-autumn, respectively^(26)^.

### Covariates

Covariates of theoretical, practical or previous empirical evidence of association with periodontitis, tooth loss and 25(OH)D level were included. Age (years) was used as a continuous variable in the statistical modelling. Sex was categorised as male/female. Body weight and height were measured using standard procedures during the health assessment. BMI was calculated as weight/height^2^ (kg/m^2^). Smoking behaviour was divided into three categories: never smoked, former smoker and current smoker. Co-morbidity variables included a doctor’s previous diagnosis of atherosclerotic CVD (yes/no), hypertension (yes/no), diabetes (yes/no) and osteoporosis (yes/no). Daily medications (including prescription, non-prescription and dietary/vitamin supplements) were also recorded and assigned WHO Anatomical Therapeutic Chemical classification codes. Specific to this analysis, vitamin D supplementation (yes/no) was included. Education was classified as primary (some primary/not complete; primary or equivalent), secondary (intermediate/junior/group certificate or equivalent; leaving certificate or equivalent) and tertiary (diploma/certificate; primary degree; postgraduate/higher degree). Socio-economic status was derived based on the respondent’s current occupation (or historic occupation – defined as the job title of the highest paying job they ever held – if they had retired). The coding of occupations followed the Irish Central Statistics Office social class schema: professional, managerial, non-manual, skilled manual, semi-skilled, unskilled and all others gainfully occupied but unknown. These were then aggregated into three categories. All covariate data utilised relates to data collected at Wave 3 (the time of the oral health assessment and blood sample acquisition).

### Statistical analysis

Baseline characteristics of the sample were summarised by quintiles of 25-hydroxyvitamin D concentration. Categorical variables were explored using *n* (%) and continuous variables by means and standard deviations. ANOVA was utilised to compare continuous variables and χ^2^ tests for categorical variables.

Multiple logistic regression analysis was performed to investigate the association between 25(OH)D concentration and the presence of periodontitis. The exposure variable, 25(OH)D concentration, was utilised both as a continuous variable (per 10 nmol/l increase) and as a categorical variable based on quintiles of 25(OH)D concentration with the highest quintile (Q5) as the reference category. A ‘crude’ regression model was carried out without adjustment for any confounding variables. A ‘fully adjusted’ regression model was subsequently carried out, which included adjustment for age (continuous variable), sex, BMI (categorised as < 24·9 kg/m^2^ (ref); 25·0–29·9 kg/m^2^; and > 30·0 kg/m^2^), diabetes, hypertension, atherosclerotic CVD, osteoporosis, smoking, education, socio-economic status and time of year bloods taken. A test for trend was performed across 25(OH)D concentration quintiles.

Multinomial logistic regression analysis was performed to investigate the association between 25(OH)D concentration and tooth loss. The exposure variable, 25(OH)D concentration, was again utilised both as a continuous variable (per 10 nmol/l increase) and as a categorical variable (quintiles). The outcome variables were based on three groups: ≥ 20 teeth (base outcome), 1–19 teeth and edentulous. Modelling again involved a ‘crude’ unadjusted model and a ‘fully adjusted’ model adjusting for all the previously aforementioned covariates. A test for trend was similarly performed across 25(OH)D concentration quintiles.

As this study was a secondary analysis based on findings from an observational study with a large sample size, a specific *a priori* power calculation was not necessary. The level of statistical significance was set at *P* < 0·05. Analyses were performed using Stata 15 (StataCorp 2017, Stata Statistical Software: Release 15, StataCorp LLC.).

## Results

The mean age of *n* 2346 participants was 65·3 years (sd 8·2), and 55·3 % of the group were female. Of the dentate subjects (*n* 2069) who had a periodontal examination, 1207 (58·3 %) had periodontitis. In terms of the number of teeth, 1292 (55·1 %) had ≥ 20 teeth, 824 (35·1 %) had 1–19 teeth and 230 (9·8 %) of the participants were edentulous.

Baseline characteristics of participants by quintile of 25(OH)D concentration are reported in Table 1. Quintile 1 = 10–45·2 nmol/l; quintile 2 = 46–60·7 nmol/l; quintile 3 = 61–73·2 nmol/l; quintile 4 = 74–89·5 nmol/l; and quintile 5 = 90–241 nmol/l. Periodontitis prevalence ranged from 66·1 % in the lowest quintile down to 49·6 % in the highest quintile, *P* < 0·001. The mean number of teeth in the lowest quintile was 16·6 (sd = 9·5) and 18·6 (sd = 8·7) in the highest, *P* < 0·01. When comparing tooth loss categories, the proportions of participants in the three groups (≥ 20 teeth, 1–19 teeth and edentulous) significantly differed across quintiles (*P* = 0·01). For example, there were proportionally a greater number of edentates in the lowest quintile compared with the highest quintile (14·0 % *v*. 9·3 %, respectively). Age was marginally but statistically significant across quintiles (*P* = 0·04), and the proportions of males/females also differed significantly over quintiles (*P* < 0·001). BMI was highest in the lowest quintile with a mean of 29·8 (sd = 5·7) and lowest in the highest quintile with a mean of 27·2 (sd = 4·6). There was a significant difference across smoking categories with greater proportions of current and former smokers in those in the lowest quintile, *P* < 0·01. There was a significant difference in the proportions of participants with a history of osteoporosis (*P* < 0·001). The greatest proportion of those with osteoporosis (24·5 %) was in the highest quintile of 25(OH)D concentration. Vitamin D supplement was highest (18·3 %) in the highest quintile concentration and lowest (5·0 %) in the lowest quintile group, *P* < 0·001. Finally, the social class structure was proportionally significantly different across quintiles, *P* < 0·001.

**Table 1.** Characteristics of cohort by 25-hydroxyvitamin D concentration, *n* 2346 (numbers and percentages; mean values and standard deviations)

			25-Hydroxyvitamin D concentrations nmol/l										
	All participants		Quintile 1		Quintile 2		Quintile 3		Quintile 4		Quintile 5		*P*
			10–45·2 nmol/l		46–60·7 nmol/l		61–73·2 nmol/l		74–89·5 nmol/l		90–241 nmol/l		
	*n*	%	*n*	%	*n*	%	*n*	%	*n*	%	*n*	%	
*n*	2346		465		467		443		485		486		
Periodontitis, *n* (%)	1207	58·3 %^a^	261	66·1 %	254	62·1 %	248	62·2 %	228	52·9 %	216	49·7 %	**< 0·001**
Number of teeth													
Mean	18·0		16·6		17·8		18·7		18·1		18·6		**< 0·01**
sd	8·9		9·5		8·6		8·5		9·1		8·7		
Tooth loss category, *n* (%)													
≥ 20 teeth	1292	55·1 %	231	49·7 %	245	52·5 %	259	58·5 %	271	55·9 %	286	58·9 %	**0·01**
1–19 teeth	824	35·1 %	169	36·3 %	178	38·1 %	153	34·5 %	169	34·9 %	155	31·9 %	
Edentate	230	9·8 %	65	14·0 %	44	9·4 %	31	7·0 %	45	9·3 %	45	9·3 %	
Age, years													
Mean	65·3		65·5		64·3		65·2		65·6		65·8		**0·04**
sd	8·2		8·8		8·0		8·4		7·9		7·8		
Sex, female, *n* (%)	1297	55·3 %	259	55·7 %	243	52·0 %	219	49·4 %	264	54·4	312	64·2 %	**< 0·001**
BMI, kg/m^2^													
Mean	28·5		29·8		29·3		28·4		27·9		27·2		**< 0·001**
sd	5·0		5·7		5·0		4·9		4·1		4·6		
Smoking status, *n* (%)													
Never	1127	48·0 %	205	44·1 %	212	45·4 %	226	51·0 %	230	47·4 %	254	52·3 %	**< 0·01**
Former	1009	43·0 %	202	43·4 %	200	42·8 %	184	41·5 %	217	44·7 %	206	42·4 %	
Current	210	9·0 %	58	12·5 %	55	11·8 %	33	7. 5 %	38	7·8 %	26	5·4 %	
Diabetes, *n* (%)	172	7·3 %	43	9·3 %	38	8·1 %	35	7·9 %	25	5·2 %	31	6·4 %	0·12
Hypertension, *n* (%)	860	36·8 %	180	38·9 %	186	40·0 %	157	35·6 %	180	37·2 %	157	32·3 %	0·12
ACVD, *n* (%)	198	8·4 %	42	9·0 %	40	8·6 %	36	8·1 %	47	9·7 %	33	6·8 %	0·56
Osteoporosis, *n* (%)	355	15·1 %	48	10·3 %	44	9·4 %	56	12·6 %	88	18·1 %	119	24·5 %	**< 0·001**
Vitamin D supplement use, *n* (%)	235	10·0 %	23	5·0 %	26	5·6 %	34	7·7 %	63	13·0 %	89	18·3 %	**< 0·001**
Education level, *n* (%)													
Primary/none	449	19·2 %	105	22·8 %	103	22·1 %	76	17·2 %	85	17·5 %	80	16·5 %	0·09
Secondary	937	40·0 %	174	37·3 %	181	38·8 %	169	38·2 %	202	41·7 %	211	43·5 %	
Third level/higher degree	959	40·9 %	186	39·9 %	183	39·2 %	198	44·7 %	198	40·8 %	194	40·0 %	
Socio-economic status, *n* (%)													
Routine, manual, other	471	20·1 %	122	26·2 %	116	24·8 %	59	13·3 %	84	17·3 %	90	18·5 %	**< 0·001**
Intermediate	995	42·4 %	187	40·2 %	181	38·8 %	199	44·9 %	210	43·3 %	218	44·9 %	
Managerial, technical and professional	880	37·5 %	156	33·6 %	170	36·4 %	185	41·8 %	191	39·4 %	178	36·3 %	

ACVD, atherosclerotic CVD. ^a^Excluding edentates/CPI score of all Xs; numbers in **bold** signify statistical significance *P* < 0·05.

**Fig. 2.**
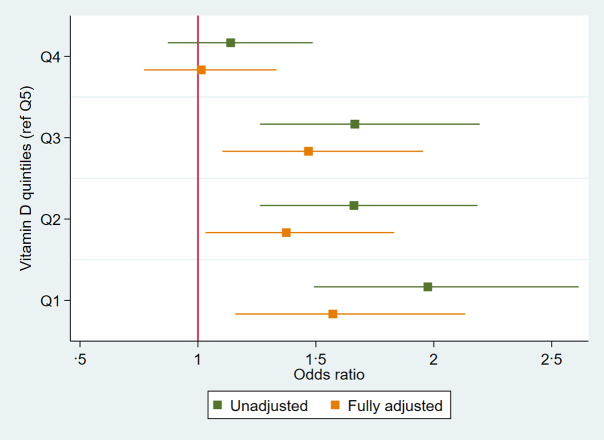
OR plot of association between 25-hydroxyvitamin D concentration per quintile (reference category Q5) and periodontitis, *n* 2060.

Multiple logistic regression was used to examine the association between 25(OH)D concentration and the presence of periodontitis (CPI 3–4) based on *n* 2060 dentate individuals (Table 2). Results indicated, after adjusting for age, sex, BMI, diabetes, hypertension, atherosclerotic CVD, osteoporosis, smoking, education, socio-economic status and time of year bloods taken, that per 10 unit increase in 25(OH)D concentration, there was a 6 % decrease in the odds of periodontitis (OR = 0·94; 95 % CI 0·91, 0·98; *P* < 0·01). When analysing, based on the quintile of 25(OH)D concentration, participants in the lowest quintile had a ×1·57 (OR = 1·57; 95 % CI 1·16, 2·13; *P* < 0·01) greater chance of periodontitis compared with the highest quintile group in the fully adjusted model. Participants in the second quintile had an OR = 1·37 (95 % CI 1·03, 1·83), third quintile OR = 1·47 (95 % CI 1·10, 1·96) and fourth quintile OR = 1·01 (95 % CI 0·77, 1·33), all compared with the highest quintile. A trend test across quintiles was significant (*P* < 0·001) suggesting a dose–response relationship as depicted in Fig. 2.

**Table 2.** Summary table of logistic regression analysis for the association between 25-hydroxyvitamin D levels and periodontitis, *n* 2060* (odds ratio and 95 % confidence intervals)

Exposure variable	Crude model			Fully adjusted model^a^		
	OR	95 % CI	*P*	OR	95 % CI	*P*
25-Hydroxyvitamin D concentration, per 10 nmol/l increase	**0·92**	**0·88, 0·95**	**< 0·001**	**0·94**	**0·91, 0·98**	**< 0·01**
25-Hydroxyvitamin D concentration, quintiles						
Q5	Reference			Reference		
Q4	1·14	0·87, 1·49	0·34	1·01	0·77, 1·33	0·91
Q3	**1·67**	**1·26, 2·19**	**< 0·001**	**1·47**	**1·10, 1·96**	**< 0·01**
Q2	**1·66**	**1·26, 2·19**	**< 0·001**	**1·37**	**1·03, 1·83**	**0·03**
Q1	**1·98**	**1·50, 2·61**	**< 0·001**	**1·57**	**1·16, 2·13**	**< 0·01**
			**< 0·001** ^**a**^			**< 0·001** ^**a**^

^a^Model adjusted for age, sex, BMI, diabetes, hypertension, atherosclerotic CVD, osteoporosis, smoking, education, socio-economic status and time of year bloods taken; *nine cases of missing covariate data (0·4 %).*P_for trend_; numbers in **bold** signify statistical significance *P* < 0·05.

**Fig. 3.**
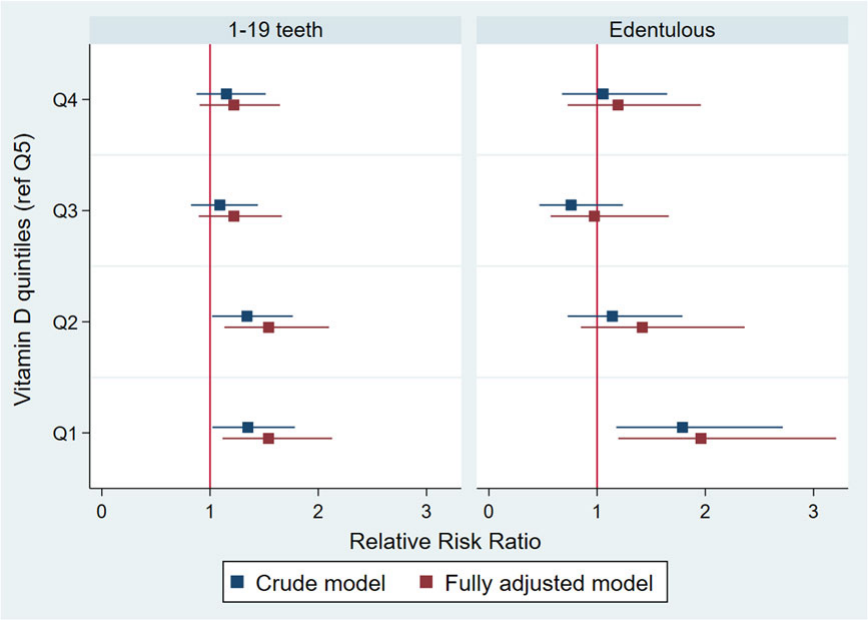
Relative risk ratio plot of association between 25-hydroxyvitamin D concentration per quintile (reference Q5) and tooth loss, *n* 2333.

Multinomial logistic regression was used to investigate the association between 25(OH)D concentration and number of teeth based on 3 groups (≥ 20 teeth (base outcome); 1–19 teeth; and Edentulous) in *n* 2333 participants (Table 3). Results indicated, after adjusting for all variables previously outlined: per 10 unit increase in 25(OH)D concentration there was a relative risk ratio reduction (RRR) of 6 % (RRR = 0·94 95 % CI (0·90, 0·98), *P* < 0·01) to have 1–19 teeth; and a RRR reduction of 10 % to be edentulous (RRR = 0·90 (95 % CI 0·85, 0·96), *P* < 0·01), relative to those with ≥ 20 teeth. When analysing based on quintiles of 25(OH)D concentration, participants in the lowest *v*. highest quintiles had a RRR of 1·55 (95 % CI 1·11, 2·13; *P* < 0·01) to have 1–19 teeth and a RRR of 1·96 (95 % CI 1·20, 3·21, *P* < 0·01) to be edentulous, relative to those with ≥ 20 teeth in the fully adjusted models (Table 3). Trend tests across quintiles were significant for both outcomes (1–19 teeth and edentulous), suggesting a dose–response relationship as observed in the RRR plots in Fig. 3.

**Table 3.** Summary table of multinomial logistic regression analysis for association between 25-hydroxyvitamin D concentration and tooth loss, *n* 2333* (relative risk ratios and 95 % confidence intervals)

Outcome variable	Exposure variable	Crude model			Fully adjusted model^a^		
		RRR	(95 % CI)	*P*	RRR	(95 % CI)	*P*
≥ 20 teeth (base outcome)							
1–19 teeth							
	25-Hydroxyvitamin D concentration, per 10 nmol/l increase	**0·96**	**0·92, 0·99**	**< 0·01**	**0·94**	**0·90, 0·98**	**< 0·01**
	25-Hydroxyvitamin D concentration, quintiles						
	Q5	Reference			Reference		
	Q4	1·15	0·87, 1·51	0·32	1·22	0·90, 1·64	0·19
	Q3	1·09	0·82, 1·44	0·55	1·22	0·90, 1·66	0·21
	Q2	**1·34**	**1·02, 1·76**	**0·04**	**1·54**	**1·13, 2·10**	**< 0·01**
	Q1	**1·35**	**1·02, 1·78**	**0·04**	**1·55**	**1·11, 2·13**	**< 0·01**
				**< 0·01** ^**a**^			**< 0·01** ^**a**^
Edentulous							
	25-Hydroxyvitamin D concentration, per 10 nmol/l increase	**0·92**	**0·87, 0·97**	**< 0·01**	**0·90**	**0·85, 0·96**	**< 0·01**
	25-Hydroxyvitamin D concentration, quintiles						
	Q5	Reference			Reference		
	Q4	1·06	0·68, 1·65	0·81	1·19	0·73, 1·96	0·48
	Q3	0·76	0·47, 1·24	0·27	0·97	0·57, 1·66	0·92
	Q2	1·14	0·73, 1·79	0·56	1·42	0·85, 2·36	0·18
	Q1	**1·79**	**1·18, 2·72**	**< 0·01**	**1·96**	**1·20, 3·21**	**< 0·01**
				**0·02** ^**a**^			**< 0·01** ^**a**^

RRR = relative risk ratio. ^a^Model adjusted for age, sex, BMI, diabetes, hypertension, atherosclerotic CVD, osteoporosis, smoking, education, socio-economic status and time of year bloods taken; *thirteen cases of missing covariate data (0·6 %); ^a^ *P*_for trend_; numbers in **bold** signify statistical significance *P* < 0·05.

## Discussion

The main finding of this cross-sectional cohort study was that in a group of older men and women from Ireland, 25(OH)D concentration was associated with both periodontitis and tooth loss. Per 10 nmol/l increase in 25(OH)D concentration, there was a 6 % reduction in the odds of having periodontitis in the fully adjusted model. Regarding tooth loss, per 10 nmol/l increase in 25(OH)D concentration, there was a reduction in relative risk of 6 % of having 1–19 teeth and of 10 % of being edentulous, compared with having ≥ 20 teeth. When considering quintiles of 25(OH)D concentration, participants in the lowest quintile had a ×1·57-fold increased odds of having periodontitis compared with the highest quintile group in the fully adjusted model. For tooth loss, participants in the lowest *v*. highest quintile had a ×1·55-fold increase in relative risk to have 1–19 teeth and a ×1·96-fold increase to be edentulous, relative to having ≥ 20 teeth. Trend tests across quintiles for both periodontitis and tooth loss categories were significant, suggesting a dose–response relationship.

In common with the findings of the present study, other studies have reported similar associations between vitamin D, periodontitis and tooth loss. Dietrich and co-workers evaluated the association between 25(OH)D_3_ concentrations and alveolar attachment loss in 11 202 subjects in the National Health and Nutrition Examination Survey, USA (1988–1994)^(27)^. In persons aged > 50 years, a significant association between 25(OH)D_3_ and attachment loss was observed, but not in younger participants. A further analysis utilising the same cohort also demonstrated that the odds of bleeding on probing was 20 % less amongst participants in the highest compared with the lowest quintile of 25(OH)D_3_^(9)^. More recently, Ebersole and co-workers conducted a study on participants of the National Health and Nutrition Examination Survey (1999–2004)^(28)^. A cross-sectional analysis was performed on 15 854 participants. Periodontitis was assessed using a partial mouth recording protocol (two randomly selected quadrants)^(29)^. Lower levels of vitamin D were consistently noted across the entire age range of patients with a greater difference seen in females with periodontitis. Regarding tooth loss, Jimenez and co-workers analysed data on 42 730 participants in the Health Professionals Follow-Up Study, USA^(12)^. A predicted plasma 25(OH)D concentration based on dietary, lifestyle and other variables was calculated and validated against plasma concentrations amongst a sub-sample. Incident tooth loss was measured by self-report from biennial questionnaires spanning 20 years. There was an inverse association across quintiles of predicted 25(OH)D score and incidence of tooth loss. In multivariable analyses, the highest quintile of the predicted 25(OH)D score compared with the lowest was associated with a 20 % lower incidence of tooth loss. Zhan and colleagues conducted a prospective cohort study to explore the association between 25(OH)D and the incidence of tooth loss in the Study of Health in Pomerania (SHIP), Germany^(30)^. 25(OH)D concentration was inversely associated with tooth loss. Following adjustment for a range of confounders, each 10 μg/l increase of 25(OH)D was associated with a 13 % decreased risk of tooth loss (RRR: 0·87; 95 % CI 0·79, 0·96; *P* < 0·01). The authors reported that the observed effect may have been partially mediated by periodontitis.

Despite the agreement in findings between the present study and previous studies, it should be acknowledged that not all studies have reported a consistent association. Therefore, the results of the present study should be interpreted with caution. Antonoglou *et al*. reported on a cross-sectional analysis of a non-smoking, diabetes-free subpopulation in the Finnish Health 2000 Survey^(31)^. Amongst 1262 participants with a mean age of 39·6 years (range 30–49), 25(OH)D did not seem to be related to periodontal pocketing or bleeding on probing. Similar findings were also reported in the Buffalo Osteoporosis and Periodontal Disease Study, USA^(32)^. 25(OH)D and 5-year change in periodontal disease measures were assessed amongst 655 postmenopausal women. No association between baseline 25(OH)D and subsequent 5-year change in periodontal disease measures was observed^(32)^. A similar negative finding in terms of baseline 25(OH)D and 5-year incidence of tooth loss was also reported in this cohort^(33)^. Recently, Li and colleagues performed an analysis on 7246 participants of the National Health and Nutrition Examination Survey 2009–2012^(19)^. A cross-sectional relationship was found between 25(OH)D and the risk of periodontitis. However, causal analysis using a Mendelian randomisation approach showed that periodontitis risk was not significantly associated with genetically increased levels of 25(OH)D, suggesting the observed association was not causal^(18,34)^.

Regarding the exposure variable, 25(OH)D concentration, our analysis utilised this both as a continuous variable (per 10 nmol/l) and as a categorical variable (quintiles across the study sample *n* 2347). Optimal concentrations of 25(OH)D in terms of periodontitis have not been defined^(35)^. Furthermore, there is likely a non-linear relationship between 25(OH)D concentration, periodontitis and tooth loss. Zhou and colleagues conducted a cross-sectional analysis of 2928 participants enrolled in the National Health and Nutrition Examination Survey (2013–2014)^(36)^. Severe periodontitis was found to have a non-linear relationship with 25(OH)D, whose inflection point was 102 (nmol/l). The authors concluded that when 25(OH)D is less than 102 nmol/l, 25(OH)D is negatively associated with severe periodontitis^(36)^. In one of the few interventional studies available, Krall and colleagues in a randomized controlled trial studied the effect of supplementation with vitamin D (700 mg/d) and Ca (500 mg/d) on tooth loss in a group of 145 > 65-year-olds over a 3-year period^(37)^. Supplementation significantly reduced the risk of tooth loss over the study observation period (OR = 0·4; 95 % CI 0·2, 0·9). 25(OH)D concentrations increased from a mean 71 to 112 nmol/l during the study^(37)^.

A methodological concern in this study may be the assessment of the outcome variable periodontitis, assessed utilising a partial mouth recording protocol on index teeth (CPI). In the present study, this was chosen for practical reasons: prior to the optional oral health assessment (Fig. 1), participants had endured a comprehensive general health assessment including anthropometric measures, cognitive assessment, cardiovascular assessment and gait analysis, as well as phlebotomy, lasting approximately 4 h. The time allocated for oral health assessments was therefore limited to 15 min, meaning that only the WHO epidemiological survey methodology^(23)^, incorporating the CPI, could be performed. It is well recognised that partial mouth recordings result in an underestimation of the prevalence of periodontal disease compared with full mouth recording protocols^(38)^. Conversely, CPI has been recognised as being well suited for identifying individuals who are periodontally healthy^(39)^. In the context of the present study, CPI was an appropriate measure to use as is based on pocket depth and more analogous to active periodontal disease. Other measures such as attachment loss have been recognised more as a measure of cumulative historic periodontal disease^(1)^, therefore not as appropriate when considering the current 25(OH)D concentration. Interestingly, since the 2018 classification of periodontal disease has been introduced^(40)^, the 2018 case definitions of periodontitis have strengthened the utility of partial-mouth recording protocols for population-based surveillance of periodontitis^(41)^. To add robustness to the study outcome, we also investigated the association with tooth loss. The objective measure of tooth loss is less subject to bias or misclassification compared with periodontitis^(42)^. Additionally, periodontitis is one of the main causes of tooth loss in older adult^(43)^ and is often considered as a proxy for the cumulative effects of periodontitis over time. However, directly linking periodontitis as the cause of tooth loss in the context of the present study is problematic as reasons for tooth loss can be multiple. In addition to periodontitis, dental caries, dental trauma, congenitally missing teeth and oral cancer may all be reasons for individuals presenting with missing teeth^(6)^.

Strengths of the study include the relatively large sample size (*n* 2346), which ranks comparatively as one of the larger studies investigating the associations between 25(OH)D concentration, periodontitis and tooth loss^(20)^. The exposure variable, 25(OH)D concentration, was comprehensively assessed using standardised methods performed at a single laboratory using the gold standard LC-MS, which helps minimise analytical variability and increases reliability. The outcome variables, periodontitis and tooth loss, were comprehensively assessed by clinical examination by a trained and calibrated dentist. A further strength relates to the cohort design of the study. Whilst other smaller studies have used case–control designs, an invitation for an oral health assessment was open to all TILDA Wave 3 participants attending the general health assessment (Fig. 1). Moreover, due to the design of the TILDA study with its main aim to investigate factors associated with population ageing, we were able to make use of a wide range of relevant data on potential confounding factors. In our statistical models, we were able to control for a large range of variables (including age, sex, BMI, diabetes, hypertension, atherosclerotic CVD, osteoporosis, smoking, education, socio-economic status, time of year bloods taken) suggesting that these factors did not account for the observed associations between 25(OH)D concentration, periodontitis and tooth loss.

Limitations of this study include, first, that the study is a cross-sectional study, and as such, it is not possible to determine whether 25(OH)D levels contributed to periodontitis and tooth loss or was a consequence of it. Although a statistically significant result was achieved after adjusting for confounders, the cross-sectional design precludes assessment of any temporal relationships between 25(OH)D, periodontitis and tooth loss. To investigate the temporality and direction of an association, the observations in this study would need to be confirmed with prospective studies. Second, there is selection bias related to the recruitment strategy of participants in the study. Although the TILDA study used a nationally representative sampling strategy, the health assessments including the oral health assessments required participants to attend dedicated research centres in Dublin. Participants who were not mobile or well enough would not likely have attended the in-person health assessments. Third, the analysis is prone to non-differential bias. 25(OH)D concentration was measured at a single time point and therefore does not reflect life-long vitamin D concentration status. Finally, in common with all observational studies, the possibility of residual confounding or the failure to account for other relevant confounders may have had some influence on the reported association between 25(OH)D levels, periodontitis and tooth loss. This may include genetic determinants of both 25(OH)D levels and periodontitis status. Vitamin D receptor polymorphisms appear to have some impact on the increasing the risk for periodontitis although this is not consistent in all populations^(44,45)^.

### Conclusion

In conclusion, in this cross-sectional study of older men and women from Ireland, 25-hydroxyvitamin D levels were associated with both periodontitis and tooth loss. These findings are important, given the high prevalence of vitamin D deficiency amongst older people in Ireland. Further, longitudinal-type studies should be aimed at specifically investigating potential causality and mechanisms involved. Additionally, given the fact that supplementation confers an overall health benefit with a low risk of toxicity or side effects, clinical trials are required to investigate whether vitamin D supplementation is of benefit in terms of prevention of periodontitis and tooth loss in the older adult population in Ireland.
